# Early impacts of a multi-faceted implementation strategy to increase use of medication treatments for opioid use disorder in the Veterans Health Administration

**DOI:** 10.1186/s43058-021-00119-8

**Published:** 2021-02-15

**Authors:** Allison M. Gustavson, Jennifer P. Wisdom, Marie E. Kenny, Hope A. Salameh, Princess E. Ackland, Barbara Clothier, Siamak Noorbaloochi, Adam J. Gordon, Hildi J. Hagedorn

**Affiliations:** 1 Veterans Affairs Health Services Research and Development Center for Care Delivery and Outcomes Research, Minneapolis Veterans Affairs Health Care System, 1 Veterans Drive, Mail Code #152, Minneapolis, MN 55417 USA; 2 Wisdom Consulting, New York, NY 10010 USA; 3 Department of Medicine, University of Minnesota, Minneapolis, USA; 4 Vulnerable Veteran Innovative PACT (VIP) Initiative; Informatics, Decision-Enhancement, and Analytic Sciences Center (IDEAS), VA Salt Lake City Health Care System, 500 Foothill Drive, Salt Lake City, UT 84148 USA; 5 Program for Addiction Research, Clinical Care, Knowledge and Advocacy (PARCKA), University of Utah School of Medicine, Salt Lake City, Utah USA; 6 Department of Internal Medicine, University of Utah School of Medicine, Salt Lake City, UT USA; 7 Department of Psychiatry, University of Minnesota School of Medicine, Minneapolis, MN 55455 USA

**Keywords:** Implementation, Facilitation, Opioid use disorder, Medication treatment, Veterans

## Abstract

**Background:**

Despite the risk of negative sequelae from opioid use disorder (OUD) and clinical guidelines for the use of effective medication treatment for OUD (M-OUD), many Veterans Health Administration (VHA) providers and facilities lag in providing M-OUD. An intensive external facilitation intervention may enhance uptake in low-adopting VHA facilities by engaging stakeholders from multiple clinical settings within a facility (e.g., mental health, primary care, pain specialty clinic, substance use disorder clinics). Our study identified pre-intervention determinants of implementation through qualitative interviews, described strategies employed during the first 6 months of intensive external facilitation, and explored patterns of implementation determinants in relation to early outcomes.

**Methods:**

Guided by the integrated-Promoting Action on Research Implementation in Health Services (i-PARIHS) framework, we interviewed stakeholders at low-adopting VHA facilities prior to external facilitation, employed a rapid qualitative analytic process, presented findings during facility visits, and collaboratively created facilitation action plans to achieve goals set by the facilities that would increase M-OUD uptake. The primary outcome was the Substance Use Disorder (SUD)-16, which is a VHA facility-level performance metric consisting of the percent of patients receiving M-OUD among those with an OUD diagnosis. We examined the relationship between pre-implementation factors and 6-month SUD-16 outcomes.

**Results:**

Across eight VHA facilities, we interviewed 68 participants. Implementation determinants included barriers and facilitators across innovation, context, and recipients constructs of i-PARIHS. Each facility selected goals based on the qualitative results. At 6 months, two facilities achieved most goals and two facilities demonstrated progress. The SUD-16 from baseline to 6 months significantly improved in two facilities (8.4% increase (95 % confidence interval [CI] 4.4–12.4) and 9.9% increase (95% CI 3.6–16.2), respectively). Six-month implementation outcomes showed that the extent to which M-OUD aligns with existing clinical practices and values was a primary factor at all facilities, with six of eight facilities perceiving it as both a barrier and facilitator. External health system barriers were most challenging for facilities with the smallest change in SUD-16.

**Conclusions:**

Early impacts of a multi-faceted implementation approach demonstrated a strong signal for positively impacting M-OUD prescribing in low-adopting VHA facilities. This signal indicates that external facilitation can influence adoption of M-OUD at the facility level in the early implementation phase. These short-term wins experienced by stakeholders may encourage continued adoption and long-term sustainability M-OUD.

**Supplementary Information:**

The online version contains supplementary material available at 10.1186/s43058-021-00119-8.

Contributions to the literature

- Intensive external facilitation enhances uptake of evidence-based practice in low-adopting facilities.
- Rapid qualitative analysis allows facilities to work collaboratively with the research team to tailor the external facilitation intervention for optimal results.
- Mixed methods analysis of early outcomes provides insight into the patterns of implementation determinants present during the initial period when momentum and barriers are likely the strongest.

## Background

Opioid use can lead to opioid use disorder (OUD), regardless of race, sex, age, or other characteristics [1]. Veterans are at a higher risk of OUD due to increased prevalence of acute and chronic pain conditions, mental health conditions, and social stressors [2]. In 2017, more than 69,000 Veterans receiving care through the Veterans Health Administration (VHA) had a diagnosis of OUD, double from 2013 [3]. The use and misuse of prescription opioids and illicit opioids have led to an increased rate of Veterans who die from opioid overdose [4]. For those who do not die from an overdose, the negative sequalae of OUD has implications for increased healthcare utilization, [5–7] risk of human immunodeficiency virus (HIV) and hepatitis C (HCV) infection, [8] higher costs to society, [9] and poorer quality of life [10].

Medication treatment for OUD (M-OUD)—including buprenorphine, methadone, and naltrexone—is strongly recommended for OUD, should be offered to all patients with OUD, and is a covered pharmacy benefit for Veterans [11, 12]. M-OUD treatment with methadone or buprenorphine is the gold-standard treatment [11]. Methadone is a full opioid agonist that has been approved for the treatment of OUD since 1972; however, methadone can only be delivered in highly regulated Opioid Treatment Programs [13, 14]. Such programs can only accommodate a small proportion of the total number of patients with OUD who would benefit from M-OUD. Buprenorphine, introduced in the USA in 2002, is a partial opioid agonist. Numerous outpatient studies have shown that sublingual buprenorphine maintenance treatment is equally effective as moderate doses of methadone [15, 16]. In addition, buprenorphine has advantages over methadone in that it has lower abuse potential and lower risk of overdose [17, 18]. Although prescribers are required to complete 8 h of mandatory training and receive a waiver to prescribe buprenorphine, it can be prescribed in office-based settings such as primary care, mental health care, and specialty care settings in addition to Opioid Treatment Programs. Thus, buprenorphine use may provide potential for increased access to treatment through multiple entry points in the healthcare system. When M-OUD is contraindicated or not acceptable to the patient, antagonist medication (naltrexone) can be considered with the greatest promise shown with injectable naltrexone administered once per month [19, 20].

The VHA is invested in improving and increasing access to M-OUD, [12, 13] yet rates of M-OUD prescribing for the population with OUD across VHA facilities range widely from 1 to 68% [3, 21–25]. VHA’s efforts with respect to M-OUD adoption have included organization-level solutions, which tend to consist of passive diffusion (e.g., through guideline development) [11]. Generally, such strategies are successful in only some locations which can lead to improvements in overall national quality indicators; however, such strategies can leave groups of low-adopting facilities—and consequently their patient populations—behind. Variability in M-OUD adoption suggests that active, targeted implementation strategies such as intensive external facilitation (e.g., coaching, mentoring) are required to promote change in clinical practice. External facilitation is a multi-faceted process by which external implementation experts work with facility stakeholders to promote interactive problem-solving and knowledge exchange that supports the adoption of evidence-based practice in the context of the targeted facility [26, 27].

We sought to determine whether employing a 12-month intensive external facilitation intervention enhances implementation of M-OUD at low-adopting VHA facilities [28]. This study focused primarily on implementation of buprenorphine due to the lower regulatory burden of prescribing buprenorphine compared to methadone and the higher level of evidence for effectiveness compared to naltrexone. The project will also encourage implementation of injectable naltrexone for cases where buprenorphine is not a viable or acceptable alternative for a particular patient. The Advancing Pharmacological Treatments for Opioid Use Disorder (ADaPT-OUD) study engaged stakeholders across multiple clinic setting within the facility (e.g., primary care, mental health, pain specialty clinic, substance use disorder specialty clinic) and included an on-site visit with the research team, active goal setting, education, M-OUD training, and monthly facilitation calls [28]. The study is based on the integrated-Promoting Action on Research Implementation in Health Services (i-PARIHS) framework, which hypothesizes that implementation success of a clinical innovation is driven by active facilitation with recipients (e.g., providers, leaders, patients) in their context (e.g., clinical setting) [29]. Prior to employing the external facilitation intervention, we conducted pre-implementation, qualitative interviews with stakeholders and then rapidly analyzed the data to inform facilitation action plans (i.e., site-specific steps and goals to increase M-OUD). Facilitation began with a site visit where the facilitators and individuals at the local facilities worked together to develop an action plan that could be executed rapidly to address barriers and leverage facilitators. Following the site visit, facilitators worked collaboratively with each site on their goals over the course of 1 year [28]. The purpose of this mixed methods study is to (1) categorize pre-implementation barriers and facilitators through use of qualitative inquiry with key stakeholders at intervention facilities, (2) describe the strategies employed during the first 6 months of intensive external facilitation, and (3) explore patterns of barriers and facilitators identified pre-implementation in the context of 6-month quantitative outcomes.

## Methods

### Mixed methods overview

The 12-month Advancing Pharmacological Treatments for Opioid Use Disorder (ADaPT-OUD) intervention is described elsewhere [28]. Briefly, for this mixed methods study, we conducted a qualitative, developmental formative evaluation of providers and leadership at low-adopting VHA facilities using semi-structured interviews prior to the intensive external facilitation intervention to identify barriers and facilitators to implementation of M-OUD. We analyzed interviews using a rapid qualitative review process [30], presented the preliminary results to the providers and leaders at each site during the site visit, and subsequently used the results to formulate an action plan with goals and corresponding strategies to achieve goals. Finally, we assessed 6-month outcomes in the context of ongoing facilitation efforts to assess for early response to the intervention. We used the Standards for Reporting Implementation Studies (StaRI) checklist to report our implementation study [31].

#### Sample

As previously described, [28] we initially selected 35 VHA facilities in the bottom quartile based on the facility proportion of OUD patients with an OUD diagnosis receiving M-OUD, reported as a percent. The goal was to have eight facilities randomly selected and invited to voluntarily participate in the intensive external facilitation intervention. If a facility declined to participate, then they were replaced by another randomly selected site. Of the 14 facilities approached, eight agreed to participate and six declined. All remaining facilities with low M-OUD adoption served as controls (*N*=27). Only intervention facilities were interviewed for qualitative data. The purpose of this manuscript is to describe the identified barriers and facilitators in relation to 6-month quantitative outcomes in intervention facilities, and therefore, we do not present quantitative data from control facilities here. Participants interviewed included leadership and clinical staff from primary care, mental health, pain specialty care, and substance use disorder (SUD) clinics.

#### Data collection and analysis

First, to assess local conditions at each site the research team conducted developmental formative evaluations through interviews with stakeholders to identify barriers and facilitators to implementing M-OUD. The i-PARIHS framework guided the qualitative interviews and identified determinants of implementation success or hindrance within the innovation, recipient, and context constructs [29]. i-PARIHS defines *innovation* as the intervention characteristics, including the complexity of the intervention, relative advantage compared to current practice or another intervention, usability, and evidence to support the intervention, including research-based evidence, clinical experience, and patient preferences. For this study, the *innovation* is M-OUD [29]. *Context* includes aspects internal to the organizational setting, such as leadership support, culture, organizational priorities, evaluation and feedback processes, and learning networks, as well as issues outside the organization, such as policy drivers and priorities, incentives and mandates, and inter-organizational networks [29]. *Recipients* includes characteristics of the people who enact and influence the implementation, such as their motivation, values and beliefs, goals, skills and knowledge, time, resources and support, local opinion leaders, collaboration and teamwork, and power and authority [29].

Research team members (HJH, HVM, MEK, PEA, HAS) conducted individual phone interviews with consented leadership and providers at each site. The first participants approached for interview were the SUD clinic directors and program coordinators, who then identified other key leaders and clinical stakeholders to approach for a voluntary interview. Stakeholders included psychiatrists, pharmacists, clinicians, and nurses across multiple clinical service lines including primary care, mental health, and pain specialty clinics. Stakeholders were not necessarily included from all clinics (primary care, mental health, pain) at every facility as interviews were targeted to those in clinics that were deemed most essential and most interested in participating in the effort. Phone interviews were recorded and transcribed, followed by a rapid analysis of data to inform the site-specific action plans (goals and strategies to achieve goals) and external facilitation intervention for each site. To rapidly analyze the data, research team members (HJH, HVM, MEK, PEA, HAS) individually read all transcribed interviews from a site and garnered a comprehensive list of barriers and facilitators. The team met to verify barriers and facilitators identified at each site and created a final site summary detailing the barriers and strengths as well as potential strategies that facilities could employ to increase M-OUD. The research team repeated this process for all intervention facilities. For a later, more comprehensive qualitative analysis, two researchers (HAS, MEK) individually categorized barriers and facilitators identified in the site summaries into the primary i-PARIHS constructs: context, innovation, or recipients. The two researchers then met to resolve conflicts with a third researcher (HJH) providing consultation as needed.

Next, the external facilitators (HJH and AJG) presented the site summaries during the in-person site visits to each clinical team (voluntary providers and leaders at each site). The collective review of the site reports allowed the clinical team to check the major identified barriers and identify any key barriers missed. Additionally, input from the clinical team was designed to promote greater feasibility of the action plan and effectiveness of selected implementation strategies. Details on the external facilitation intervention are described elsewhere [28]. Briefly, site visits were scheduled over 1–2 days depending on the educational needs of the site. Consistent components of facilitation included at all site visits were review of the site report as a group, education for the clinical team on M-OUD topic areas and available resources, and engagement of the clinical team in identifying goals and strategies to achieve goals. Site visits were staggered over a 15-month period, beginning in March of 2018 and ending June of 2019. Site visits were followed by 12 months of facilitation, including monthly coaching calls, ending in July of 2020. All facilities had completed a minimum of 6 months of the intervention prior to the COVID pandemic. Researchers (HJH and MEK) reviewed progress notes gathered from the first 6 months of telephone meetings with the facilities to assess whether goals were achieved. If the site indicated the goal was accomplished by the 6-month call, the goal was considered accomplished (“Yes”). If significant progress had been made toward the goal, it was indicated as “In progress.” If no progress or very little progress had been made, the goal was not considered accomplished (“No”).

Finally, we investigated the relationship between barriers and facilitators with the primary outcome of the SUD-16 metric, which is a VHA metric defined by clinical operations that indicates the percent of patients with an OUD diagnosis receiving M-OUD treatment (formulations of methadone, buprenorphine, naltrexone). These patients with OUD are served by the facility and its associated community-based clinics in the past year. The SUD-16 metric is reported quarterly for each fiscal year. The SUD-16 for the fiscal year and quarter just prior to the facility visit date and any prior interviews was used as baseline data. The SUD-16 for the fiscal year and quarter ending about 6 months after the on-site facility visit was used for 6-month data (last site December 31, 2019). We analyzed the facility change in SUD-16 from baseline to 6 months using independent two sample *z* tests, since we did not have patient level data to assess the extent of dependency between the two periods. We reported 95% confidence intervals. Statistical significance was determined at *p*<0.05. Because the true correlation between baseline and 6 months is unknown, but assumed positive, the above test yields larger standard errors and hence larger *p* values implying the reported *p* values are conservative.

## Results

We interviewed 68 out of the 85 stakeholders approached for participation, resulting in an 80% completion rate (3 refusals and 14 unable to schedule interviews before facility visit). Table 1 summarizes VHA facility characteristics and the roles of interviewed stakeholders (*N*=68, range 7–11 per facility). Each facility had at least three interview participants each in the leadership and clinical provider categories. The facilities represented diverse geographical locations with two out of the 8 facilities classified as geographically located in rural areas [18]. While six of eight facilities were categorized as “urban,” only one of these was in a major metropolitan area.

**Table 1:** Number and type of staff interviewed at each VHA facility and facility characteristics

Facility #	Clinical providers (***N***)	Leaders (***N***)	Clinic settings represented	Region	Urban or rural
**Facility 1**	3	6	Pharmacy, Pain Specialty Clinic, Mental Health, Primary Care, Inpatient, SUD	Rocky Mountain	Rural
**Facility 2**	3	4	Pharmacy, SUD, Mental Health	Rocky Mountain	Urban
**Facility 3**	3	5	Pharmacy, Satellite Clinic, Primary Care, Inpatient	Southeast	Rural
**Facility 4**	7	4	Pharmacy, Mental Health, SUD, Primary Care	Northwest	Urban
**Facility 5**	3	4	Pharmacy, Mental Health, Primary Care, Pain, SUD	Northcentral	Urban
**Facility 6**	3	6	Pharmacy, SUD, Pain, Mental Health	Southwest	Urban
**Facility 7**	3	5	Pharmacy, Pain, Mental Health, SUD	Southeast	Urban
**Facility 8**	6	3	Pharmacy, Mental Health, Pain, SUD	Northcentral	Urban

### Study Question 1: What are barriers and facilitators identified by VHA facilities?

Table 2 describes examples of barriers and facilitators within selected, pre-defined i-PARIHS categories of innovation, context, and recipients. It is possible for a construct to be both a barrier and a facilitator, even within the same facility. The innovation construct’s sub-element “degree of fit with existing practice and values” is one example. Many clinicians endorsed the need to manage patients stabilized on buprenorphine in their clinic (facilitator), yet they often had trouble identifying which patients with OUD on their panels might be appropriate to manage outside of a substance use disorder specialty care setting (barrier). We chose to illustrate a few key barriers and facilitators that were common across all VHA facilities and frequently integrated into many of the facility-specific action plans [22–24]. Innovation barriers and facilitators included degree of fit with existing practice and values, clarity, and relative advantage. In terms of degree of fit, some clinicians mentioned processes already in place regarding where and when to treat patients with M-OUD that would help facilitate increased prescribing and management of patients with M-OUD. Meanwhile, at other facilities, the current processes for approving an M-OUD prescription were extensive and thus clinicians cited this as a barrier to M-OUD implementation. Overall, clinicians at many facilities perceived a lack of clarity in policies and processes regarding who treats patients with M-OUD outside of the specialty SUD clinics. To match the high demand for M-OUD in Veterans, clinician and leadership stakeholders perceived the relative advantage of providing M-OUD throughout the VHA over existing practices limiting the provision of these services to within SUD specialty care clinics.

**Table 2:** Examples of barriers and facilitators described by facilities and classified by i-PARIHS constructs

	i-PARIHS construct	Barrier example	Facilitator example
**Innovation** (focus or content of innovation effort)	Degree of fit with existing practice and values	Approval for buprenorphine is difficult given current requirements for 4 positive consecutive urine drug screens for opiates and no other substances	An established pain clinic has a current role in identifying patients with OUD, initiating buprenorphine, and transferring care back to primary care
	Clarity	M-OUD treatment is referred to community clinics (non-VHA providers), so roles and responsibilities of VHA staff regarding M-OUD is not clear	Facility currently has clear criteria that patient misuse or diversion (selling) of buprenorphine results in loss of medication eligibility
	Relative advantage	No barriers reported	Non-VHA community clinics can have long wait lists and may not accept VHA insurance, which provides a relative advantage for VHA facilities to treat and manage patients on M-OUD
**Context** (multiple layers that can facilitate or constrain implementation)	Local level: Mechanisms for embedding change	Clinic staff attempted to remove restrictions on where buprenorphine initiations could occur (currently only in the emergency room in a separate building); however, they were met with resistance due to concerns for adverse events related to buprenorphine initiation	Facility previously identified team to clarify and implement buprenorphine initiation strategies to increase access
	Organizational level: Organizational priorities	VHA employs multiple initiatives to improve Veteran care, which often leads to competing priorities	Organizational leadership is aware SUD-16 metric is low and are requesting improvement
	External Heath System Level: Environmental stability	Rurality of facility brings the challenge of maintaining an adequate number of trained and waivered staff to provide adequate coverage	No facilitators reported
**Recipients** (people who enact and influence the implementation)	Motivation	Limited interest from non-SUD disciplines and clinics to manage patients on buprenorphine	Providers want to get the necessary training to prescribe buprenorphine
	Skills and knowledge	Facility does not have a provider who is waivered to prescribe	Facility has a new provider with experience in a buprenorphine clinic
	Time, resources, and support	Lack of adequate space and provider capacity to prescribe buprenorphine and manage an increase in patient caseload	Mental health residential programs have staff who can be re-allocated to provide buprenorphine and manage patients

Context barriers and facilitators were spread across local, organizational, and external health system levels. Overall, stakeholders described more facilitators than barriers at the local level with the most frequently mentioned being local level mechanisms for embedding change, meaning stakeholders reported positive perceptions of the facility’s past experience with employing processes to enact practice change. At the organizational level, stakeholders perceived that facility-level leadership was promoting a strong call to action to prioritize improvement of SUD-16 metrics but may have been inundated by the multiple VHA initiatives concurrently in place. At the external health system level, environmental stability was affected by the rurality of some facilities which subsequently impacted leadership’s ability to recruit and maintain trained and waivered providers.

Recipient-related barriers and facilitators included motivation, skills, knowledge, time, resources, and support. Stakeholders perceived that providers at low-adopting VHA facilities wanted to complete the required training to apply for the United States Drug Enforcement Administration (DEA) waiver necessary to prescribe buprenorphine but also indicated not all providers had a shared interest depending on the provider’s team or clinic. For example, primary care providers often expressed concerns with large caseloads and limited resources as a reason for not expanding access to buprenorphine to their clinics. Citing skills and knowledge as a determinant of buprenorphine implementation depended on whether the facility already employed a provider who possessed a DEA waiver and had experience prescribing buprenorphine. Adequate space and provider capacity (number of providers, caseload per provider, and number of additional staff) were perceived as important barriers or facilitators to implementation of buprenorphine (Table 2).

### Study Question 2: What strategies were employed as part of the intensive external facilitation intervention?

Table 3 describes the facility-selected goals and strategies employed as part of the intensive external facilitation strategy during the initial 6 months following the on-site, facility visit. Facilities identified a range of 3–5 goals. Goals were related to expanding capacity of clinics to prescribe, implementing buprenorphine prescribing across different clinics (i.e., residential mental health, outpatient mental health, primary care, SUD clinics, and pain clinics), increasing access through different mechanisms of care delivery (e.g., telehealth), or by transferring care after initiation to other providers (e.g., primary care, mental health) for maintenance. For each goal, facilities and external facilitators worked together to establish 1–4 strategies to accomplish the identified goal. Strategies included identifying education topics, increasing the number of DEA waivered prescribers through provision of required training and facilitation of the waiver application process, identifying teams or providers available to deliver care management to patients on buprenorphine, determining criteria for monitoring of patients on buprenorphine, investigating incentive options for providers to increase buprenorphine prescribing, and revising protocols regarding where buprenorphine initiations can take place and which providers should be involved. Facilities had varying degrees of success in achieving their goals, ranging from zero to almost all goals achieved in the first 6 months (Table 3).

**Table 3:** Implementation strategies within the external facilitation intervention used to address barriers or leverage facilitators to M-OUD at low-adopting VHA facilities

Facility	Goals	Strategies identified	Achievement of goals
**Facility 1**	Implement buprenorphine prescribing in outpatient mental health clinic	1. Identify temporary care management staffing prior to new nurse hire 2. Ensure prescribing privileges for all relevant outpatient mental health prescribers 3. Create scheduling mechanisms for outpatient mental health providers to treat patients with buprenorphine	Yes
	Implement buprenorphine prescribing in pain clinic	1. Provide waiver training and privileging for prescribers 2. Pharmacy to assist with care coordination to reduce burden on providers	In progress
	Implement buprenorphine prescribing in residential mental health program	1. Create templates in the electronic medical record to facilitate accurate documentation of buprenorphine visits 2. Provide waiver training and privileging for prescribers	Yes
	Educate primary care providers on buprenorphine	1. Schedule seminar or cyber seminar with national subject matter expert 2. Develop and present medical grand rounds with local subject matter expert	Yes
**Facility 2**	Conduct pilot to transfer one patient on buprenorphine to another waivered provider outside of SUD for maintenance monitoring	1. Determine criteria providers would accept for stable patients on buprenorphine to transfer back into their care (e.g., number of consecutive clean urine screens)	No
	Examine M-OUD policies to maximize patient eligibility and ensure safety needs are addressed	1. Provider to review other VHA policies, revise current policy, and send to subject-matter expert for review	No
	Identify incentives to encourage providers to obtain waivers and treat patients with buprenorphine-	1. Investigate incentive options for potential prescribers	In progress
	Implement pharmacy care management model for patients on buprenorphine in primary care	1. Identify pharmacy lead 2. Assess willingness of waivered primary care provider to prescribe buprenorphine and hand-off management to pharmacy	In progress
**Facility 3**	Develop outpatient buprenorphine clinic in mental health clinic	1. Determine whether provider can have a half day clinic in the main mental health clinic	Yes
	Provide buprenorphine initiations in pain clinic that was soon to be established	1. Form a team of providers focused on treating pain who are willing and have capacity to provide buprenorphine initiations	No
	Identify primary care and mental health providers who are willing to have stable patients on buprenorphine returned to them from community providers to prescribe in house	1. Identify providers who are willing and have the capacity to manage patients on buprenorphine in caseload	Yes
	Implement buprenorphine prescribing in VHA satellite clinic	1. Identify actionable steps for making progress in prescribing buprenorphine in a VHA satellite clinic	No
**Facility 4**	Implement buprenorphine prescribing in a VHA substance abuse treatment program	1. Provider to complete waiver training to prescribe 2. Renew community contracts for one more year to allow new prescriber to bring patients on M-OUD back to VHA gradually	Yes
	Develop mechanism to transfer stable patients on buprenorphine from VHA substance abuse treatment program back to VHA mental health or primary care providers	1. Identify mental health and primary care providers that are waivered and willing to oversee management of patients on buprenorphine already on their caseload. 2. Identify mental health and primary care providers who are waivered and willing to take patients on buprenorphine who are not already on their caseload. 3. Identify additional training or resources that these providers would need to feel comfortable managing patients on buprenorphine	No
	Implement buprenorphine prescribing in primary care through a team focused on pain	1. Identify resources to prescribe buprenorphine	Yes
**Facility 5**	Implement incentives for providers to become credentialed and prescribe	1. Request financial incentives from mental health regional leadership	Yes
	Expand buprenorphine prescribing in mental health	1. Increase number of waivered, credentialled prescribers in clinic	No
	Expand buprenorphine prescribing to a pain clinic	1. Increase number of waivered, credentialled prescribers in clinic	Yes
	Identify options for transferring stable patients back to primary care	1. Determine criteria providers would accept for transfer of stable patients on buprenorphine back into their care (e.g., number of consecutive negative urine screens)	No
	Provide initiations in the Mental Health residential program	1. Identify education and observation opportunities for clinic staff to increase comfort with initiations	No
**Facility 6**	Implement buprenorphine prescribing in pain clinic	1. Hire an addiction specialist to increase capacity to prescribe	No
	Implement buprenorphine prescribing in mental health same day access clinic	1. Determine pharmacy processes for buprenorphine dispensing for same day access 2. Evaluate implementation of warm hand offs from mental health same day access clinic to general mental health clinic one month after initiation for patients on buprenorphine 3. Identify nurse champion who can follow-up with patients weekly while in access clinic to support other providers and nurses during buprenorphine stabilization phase 4. Identify provider available as interim coverage for patient education on buprenorphine side-effects, obtain consent for treatment, register patients in the state prescription drug monitoring program, and provide naloxone kits	In progress
	Implement buprenorphine in care teams focused on homeless Veterans	1. Increase number of waivered prescribers in clinic	No
	Increase access to buprenorphine maintenance through telehealth	1. Utilize waivered prescriber to administer telehealth for stable mental health patients on buprenorphine who live far from clinic	No
	Minimize local restrictions on buprenorphine prescription access	1. Remove requirement for providers to receive pharmacy approval prior to prescribing buprenorphine	No
**Facility 7**	Implement buprenorphine prescribing in pain clinic	1. Hire a part-time psychiatrist or an addiction fellow in the pain clinic to prescribe buprenorphine for patients with pain and OUD 2. Obtain approval from pharmacy to allow physicians with a waiver in the pain clinic to prescribe buprenorphine for patients with pain and OUD	In progress
	Implement buprenorphine prescribing in behavioral health and outpatient substance abuse clinic	1. Increase buprenorphine prescribing by currently waivered providers in both clinics 2. Increase number of waivered providers in behavioral health clinic to increase capacity to prescribe	Yes
	Implement M-OUD prescribing in community-based outpatient clinics	1. Consult with nursing leadership about nurses assisting telehealth mental health providers in community-based outpatient clinics 2. Consult with nursing leadership about offering naltrexone extended release in community-based outpatient clinic 3. Establish a telehealth program in the mental health clinic and have a part-time psychiatrist prescribe buprenorphine for patients with OUD	In progress
	Implement buprenorphine prescribing in primary care	1. Create lists of actionable OUD patients by provider to give physicians information regarding the number of patients in their caseload who may be appropriate M-OUD. 2. Identify incentives to encourage providers to obtain waivers and treat patients with buprenorphine within the primary care mental health integration clinic, establish a part-time, SUD, non-prescribing provider for SUD assessment, and a psychiatrist for prescribing buprenorphine 3. Create a primary care-based treatment team, with embedded mental health, to prompt increased buprenorphine prescribing	No
**Facility 8**	Expand locations for initiation of buprenorphine by removing local restrictions	1. Meet with nursing leadership to discuss removing local restrictions that initiations must occur in the emergency room 2. Revise standard operating procedure to remove local restrictions on where initiations can occur 3. Meet with pharmacy to discuss mailing buprenorphine to allow clinics without pharmacies to initiate buprenorphine	Yes
	Streamline process for approval for initiation for faster patient access to buprenorphine	1. Determine which steps are essential in the current process of reviewing patients during a weekly committee meeting and which can be removed to expedite access to treatment	Yes
	Implement buprenorphine prescribing in residential substance use treatment program	1. Reach consensus on strategy to time buprenorphine initiation either prior to admission to the residential program or at discharge from the residential program	Yes
	Increase number of prescribers in primary care	1. Identify incentives to encourage providers to obtain waivers and treat patients with buprenorphine 2. Develop nurse care management model with nursing leadership. 3. Establish criteria for patients on buprenorphine to return to primary care provider for maintenance	No

### Study Question 3: How did the pattern of the pre-implementation facilitators/barriers impact early 6-month outcomes?

Table 4 displays the quantitative data with the SUD-16 at baseline and 6 months post facility visit and the difference (6-month baseline). Baseline SUD-16 percent proportions ranged from 4.0% to 34.9%. Seven out of eight facilities improved on their SUD-16 at a range of 4.3 to 9.9%, with two facilities demonstrating statistically significant increases (facilities 4 & 5). Facility 6 had the largest baseline SUD-16 at 34.9% and was the only facility to experience a negative difference (− 3.6%), though not statistically significant (Table 4).

**Table 4:** SUD-16 measure at baseline and 6 months following initial facility visit, ranked ordered by the magnitude of within-facility difference

	SUD-16 at baseline^a^ (%)	SUD-16 at 6 months post facility visit^a^ (%)	Difference in SUD-16 (6-month baseline) (%)	95% CI^b^
**Facility 6**	34.9	31.3	− 3.6	(− 9.5 to 2.2%)
**Facility 1**	4.0	7.4	3.4	(− 4.4 to 12.4%)
**Facility 7**	18.8	23.1	4.3	(− 0.7 to 9.3%)
**Facility 3**	10.4	17.3	6.9	(− 0.7 to 14.5%)
**Facility 8**	28.5	35.6	7.1	(− 0.4 to 14.5%)
**Facility 2**	15.6	23.3	7.7	(− 2.7 to 18.2%)
**Facility 4**	4.1	12.5	8.4	(4.4 to 12.4%)^‡^
**Facility 5**	19.2	29.1	9.9	(3.6 to 16.2%)^‡^

^a^Quarterly SUD-16 measures

^b^Without patient level data, independent percent proportions are assumed at each time point

^‡^Statistically significant at *p* < 0.05

The supplementary material provides a visual representation of the mixed methods results representing the patterns of pre-intervention barriers and facilitators in the context of the quantitative SUD-16 change from baseline to 6 months. Under innovation, the extent to which buprenorphine prescribing aligned with existing clinical practices and values (i.e., degree of fit) was present as a barrier, facilitator, or both at all facilities prior to the intervention, with little pattern noted in respect to the SUD-16 change. The lack of clarity in roles and procedures around buprenorphine prescribing was noted as a barrier in the facilities with the greatest change in SUD-16. Under recipients, the only construct that showed a pattern consistent with quantitative outcomes was skills & knowledge (i.e., possessing the information and ability to implement buprenorphine) with four of the lowest performing facilities identifying it as a barrier and two of the highest performing facilities identifying it as a facilitator. Under context, barriers at the wider healthcare and regulatory policy level (i.e., external health system) were present in the six facilities with lowest rank-order change in SUD-16. The two facilities with the least change in SUD-16 indicated a higher number of facilitators at the local VHA-facility level compared to the other six facilities.

## Discussion

The results of this study achieved our purpose by identifying pre-intervention barriers and facilitators to implementation of M-OUD in low-adopting VHA facilities, illustrating the strategies used during the external facilitation intervention to achieve facility-specific goals, and generating hypotheses regarding the pattern of barriers and facilitators in the context of 6-month quantitative outcomes.

First, the nature of the pre-intervention barriers found in this study were similar to other studies exploring factors impeding VHA-wide uptake of M-OUD including scarce availability of prescribers, limited staff training, inadequate space, patient and provider interest, or lack thereof [22–24, 27]. Our study found that facility goals focused on reducing barriers rather than leveraging the positive impact of facilitators. However, facilities may choose to address specific barriers that are conducive to concurrently leveraging a facility’s facilitators. For example, a facility may select a goal to implement M-OUD in mental health (to address a gap) by leveraging the collaborative teamwork already in place. Future research is needed to explore the interaction between barriers and facilitators to optimize facilitation strategies and tailor to specific facility needs.

Second, the results of the facility-specific facilitation action plan at 6 months were variable with facilities ranging from achieving or progressing towards most goals to facilities that did not achieve or demonstrate progress towards goals. These varying results indicate some barriers (e.g., staff shortages, credentialing restrictions) may be too complex to overcome by facilitation alone over the course of 6 months. Thus, acknowledging and discussing modifiable versus non-modifiable barriers as part of the external facilitation plan is critical to working collaboratively with facilities to develop informed and feasible goals. Additionally, integration of buprenorphine within other clinics that have not traditionally prescribed it (e.g., primary care, mental health, pain specialty clinic) may not be achieved early in implementation; however, the external facilitation likely lays the groundwork and subsequent momentum to expand buprenorphine prescribing to other clinics outside of substance use disorder specialty clinics. The removal of “silos” through multi-level integration of care is important for implementation of buprenorphine since patients can potentially be identified for appropriate treatment and managed across multiple disciplines (e.g., mental health, primary care, pain specialty clinics, community-based services) [21]. Future efforts are needed to address broader issues such as insufficient workforce capacity, variation in state policies regarding M-OUD, and paucity of clinical guidelines for transferring patient care across disciplines and clinics.

Third, the mixed methods results regarding the determinants of M-OUD implementation in the context of the external facilitation intervention and 6-month quantitative outcomes generate more hypotheses regarding the patterns of barriers and facilitators that may promote or hinder the adoption of buprenorphine. For innovation, the degree of fit with existing practice and values was perceived as an important construct to address with the external facilitation to improve adoption. Understanding current processes and how changes in the present conditions to increase buprenorphine adoption may be more or less disruptive to the local environment can inform the external facilitation intervention and, ultimately, drive outcomes.

For context, the salient pattern was the presence and number of barriers in the constructs related to the wider healthcare system and regulatory policies (i.e., external health system). As such, facilities with barriers at the external health system level may demonstrate limited capacity to respond to the external facilitation because overcoming these may be a prerequisite to improving adoption of buprenorphine at the local level. A second noted pattern was rather counterintuitive, showing the two facilities with the least change in SUD-16 perceived a higher number of facilitators at the local level compared to the other six facilities. Drawing upon the discussion in Hagedorn & Heideman’s work implementing hepatitis services in SUD treatment, [32] perhaps stakeholders at facilities with more perceived contextual facilitators prior to the external intervention became quickly frustrated when experiencing setbacks during the initial efforts to increase M-OUD prescribing [32–35]. Whereas facilities with a defensive pessimistic view set low expectations for performance and anticipated setbacks, thereby taking action to prepare for or manage barriers to increasing use of M-OUD [32–35].

For recipients, barriers related to skills and knowledge and values and beliefs appeared addressable through external facilitation, as reflected in the strategies and goal achievement in the facility-specific action plans. Most stakeholders perceived motivation as a facilitator, which suggests willingness to engage in M-OUD adoption. However, despite the presence of motivation, these facilities were considered low-adopting per the baseline SUD-16 measure and thus needed a more active approach to implementation of M-OUD, hence the external facilitation intervention. Interestingly, only a few facilities indicated collaboration and teamwork was either a facilitator and barrier; however, all facilities focused at least one goal on implementing buprenorphine in a clinic or with a provider team that had not previously prescribed buprenorphine or taken on the management of patients on buprenorphine (e.g., primary care, mental health). Perhaps, the issue of collaboration and teamwork was unmasked during the facility visit with external facilitators (after the provider interviews) as cross-clinic teams worked together to strategize ways of increasing adoption of buprenorphine. A logical step identified for several facilities was to expand the number of DEA-waivered prescribers across disciplines (e.g., mental health, primary care) and increase inter-clinic provider capability and capacity to manage patients on buprenorphine. Thus, during the facility visit and subsequent 6 months of external facilitation, facilities may have encountered barriers or facilitators to teamwork and collaboration that they had not considered prior to the facility visit.

This study is unique in that we used rapid qualitative analysis of pre-implementation interviews with stakeholders regarding determinants of M-OUD implementation to inform and quickly tailor the external facilitation intervention to fit facility-level needs. Academic detailing is an effective educational outreach strategy commonly used to impact care in this patient population by targeting uptake of evidence-based practice at the provider level [30, 36]. Academic detailing [30, 36] and other approaches [21, 37] designed to promote uptake of evidence-based practice could benefit from this rapid, developmental approach to tailoring implementation strategies to fit the complex and dynamic interaction between the clinical innovation (M-OUD), recipients, and context. Our mixed methods analysis of 6-month outcomes helps us to understand whether implementation interventions and strategies can make meaningful change in facility metrics quickly to, ultimately, impact patient outcomes. This study has several limitations. First, the study was conducted in VHA facilities, all of which volunteered to participate after initial selection, which limits the generalizability; however, perhaps because these facilities were considered low-adopting facilities per their SUD-16 measure (lowest quartile in the SUD-16 measure among all VHA facilities), many were eager to participate. Like other facility metrics, SUD-16 is a facility performance metric where facility and regional leaders receive compensation based on facility performance. Second, interview participants volunteered to participate, and thus, the information gathered may be biased compared to a sample of providers who were randomly selected to provide qualitative data. To address this, we carefully crafted the interview guide to include questions and probes that sought to understand the facility’s culture and perspectives regarding M-OUD, not just the interviewee’s perspective alone. General sampling rather than a random sample for qualitative inquiry targets key stakeholders to ensure the perspectives of those involved in and/or impacted by the implementation effort are gained [38]. Third, qualitative data was collected at one time-point, prior to the external facilitation, and may not capture barriers or facilitators that occurred during the first 6 months of external facilitation intervention, such as staff turnover. We are collecting qualitative data after the full year of external facilitation, which—juxtaposed on the pre-intervention data—will likely capture changes in patterns of facilitators and barriers that may impact study results.

The full effect of the intensive external facilitation on change in M-OUD prescribing at low-adopting VHA facilities will be observed at the completion of the parent ADaPT-OUD study (12 months following facility visits) [28]. Early results from our study can assist other implementation efforts to increase adoption of M-OUD in the VHA by identifying patterns of barriers and facilitators more or less conducive to change in uptake of M-OUD [21]. Future research will explore patterns of barriers or facilitators and the facilitation response over the course of the 12-month external facilitation intervention to determine how the types, sequence, and timing of implementation strategies impacted M-OUD adoption a the various facilities.

## Conclusions

Our study addresses the variability in delivery of M-OUD across the VHA and is aligned with current VHA and non-VHA initiatives to address the opioid crisis. Over the initial 6 months of the study, intensive external facilitation to promote accomplishment of facility goals demonstrated a signal for positively impacting M-OUD prescribing in almost all low-adopting VHA facilities. The signal in our mixed methods analysis of 6-month outcomes indicates change at the facility level can occur during the initial external facilitation intervention phase. Changes in facility metrics allow stakeholders to experience short-term wins that may strengthen the long-term sustainability of M-OUD prescribing. Accelerating the implementation of M-OUD at low-adopting VHA facilities is time-sensitive given the increasing prevalence of OUD and the negative societal and individual implications associated with OUD. Enhancing adoption and sustainability of M-OUD is critical to optimizing positive outcomes for Veterans and non-Veterans with OUD across the nation.

## Supplementary Information

**Additional file 1: Table 1.** Patterns of barriers and facilitators coded within the i-PARHIS framework from facility summaries, ranked by the magnitude of within-facility difference in SUD-16.

## Data Availability

Deidentified data can be obtained by contacting the corresponding author.

## Abbreviations

ADaPT-OUD: Advancing Pharmacological Treatments for Opioid Use Disorder
DEA: Drug Enforcement Agency
i-PARIHS: integrated-Promoting Action on Research Implementation in Health Services framework
M-OUD: Medication for opioid use disorder
OUD: Opioid use disorder
SUD: Substance use disorder
SUD-16: VHA performance metric of the percent of patients receiving M-OUD out of all patients with OUD diagnosis
VHA: Veterans Health Administration
