# Supplementary material for: Early impacts of a multi-faceted implementation strategy to increase use of medication treatments for opioid use disorder in the Veterans Health Administration
Source: Implement Sci Commun. 2021 Feb 15;2:20. doi: 10.1186/s43058-021-00119-8 (PMC7885503; doi:10.1186/s43058-021-00119-8)
Supplement: Supplementary file 1 — Additional file 1: Table 1. Patterns of barriers and facilitators coded within the i-PARHIS framework from facility summaries, ranked by the magnitude of within-facility difference in SUD-16. [file 43058_2021_119_MOESM1_ESM.docx]

**Table 1.** Patterns of barriers and facilitators coded within the i-PARHIS framework from facility summaries, ranked by the magnitude of within-facility difference in SUD-16.

|  | **Facility 6** | **Facility 1** | **Facility 7** | **Facility 3** | **Facility 8** | **Facility 2** | **Facility 4** | **Facility 5** |
| --- | --- | --- | --- | --- | --- | --- | --- | --- |
| **SUD-16 % change from baseline to 6 months** | -3.6% | 3.4% | 4.3% | 6.9% | 7.1% | 7.7% | 8.4% | 9.9% |
| **Innovation** |  |  |  |  |  |  |  |  |
| Clarity |  |  |  |  |  | B | B | B+F |
| Degree of fit with existing practice and values | B+F | B+F | B+F | B+F | B+F | F | B+F | B |
| Relative Advantage |  |  |  | F | F | F | F |  |
| **Context** | | | | | | | | |
| Local Level Formal and informal leadership support | F | F |  | F |  |  |  | F |
| Local Level: Culture | F | F |  | B |  |  |  |  |
| Local Level: Mechanisms for embedding change | F | F |  | B | B+F |  | B | F |
| Local Level: Past experiences with innovation and change | F | F |  |  |  |  |  |  |
| Local Level: Evaluation and feedback processes |  |  | B |  |  |  |  |  |
| Local Level: Learning environment |  |  |  |  | F |  |  |  |
| Organization Level: Organizational priorities | F | F | F |  |  | B | F |  |
| Organizational Level: Senior leadership and management support |  |  |  |  | F | F | F |  |
| Organizational Level: Culture |  |  | B |  |  |  |  | B+F |
| Organizational Level: Structure and systems |  |  |  | B | F |  | B | B+F |
| Organizational Level: Learning networks |  |  |  |  |  |  |  | B |
| External Health System Level: Policy drivers and priorities | B+F | B+F |  | F |  | F |  | F |
| External Health system Level: Incentives and mandates |  |  | B |  | F |  |  |  |
| External Health System Level: Regulatory frameworks |  |  |  | B | B |  |  |  |
| External Health System Level: Environmental stability | B | B |  |  |  | B |  |  |
| **Recipients** | | | | | | | | |
| Motivation | F | F | B+F | F | F | F | F |  |
| Values and beliefs | B+F | B+F | B+F | B+F | B+F | B+F | B+F | B+F |
| Skills and knowledge | B | B |  | B | B | F |  | F |
| Time, resources, support | B+F | B+F | B+F |  | B | B | B+F | B+F |
| Local opinion leaders |  |  |  | B |  |  |  |  |
| Collaboration and teamwork |  |  |  | F |  | B+F | B+F |  |
| Existing networks | F | F |  | F | B+F |  |  | B+F |
| Presence of boundaries |  |  |  |  |  | B | B |  |
| **Other** | | | | | | | | |
| Staff turnover |  |  |  | B+F |  | B |  |  |
| Patient’s experience with receiving care |  |  |  |  | F | B |  |  |

Note: B=Barrier; F=Facilitator; B+F=Barrier and Facilitator
